# Surrogate vascular input function measurements from the superior sagittal sinus are repeatable and provide tissue-validated kinetic parameters in brain DCE-MRI

**DOI:** 10.1038/s41598-022-12582-x

**Published:** 2022-05-24

**Authors:** Daniel Lewis, Xiaoping Zhu, David J. Coope, Sha Zhao, Andrew T. King, Timothy Cootes, Alan Jackson, Ka-loh Li

**Affiliations:** 1 Department of Neurosurgery, Manchester Centre for Clinical Neurosciences, Salford Royal NHS Foundation Trust, Manchester Academic Health Science Centre, Manchester, UK; 2 Geoffrey Jefferson Brain Research Centre, University of Manchester, Stott Lane, Salford, M6 8HD Greater Manchester UK; 3 Division of Neuroscience and Experimental Psychology, Faculty of Biology, Medicine and Health, School of Biological Sciences, University of Manchester, Manchester, UK; 4 Division of Informatics, Imaging and Data Sciences, Wolfson Molecular Imaging Centre (WMIC), University of Manchester, Manchester, UK; 5 Division of Cardiovascular Sciences, Faculty of Biology Medicine and Health, School of Medical Sciences, University of Manchester, Manchester, UK

**Keywords:** Magnetic resonance imaging, CNS cancer, Brain, Diagnostic markers

## Abstract

Accurate vascular input function (VIF) derivation is essential in brain dynamic contrast-enhanced (DCE) MRI. The optimum site for VIF estimation is, however, debated. This study sought to compare VIFs extracted from the internal carotid artery (ICA) and its branches with an arrival-corrected vascular output function (VOF) derived from the superior sagittal sinus (VOF_SSS_). DCE-MRI datasets from sixty-six patients with different brain tumours were retrospectively analysed and plasma gadolinium-based contrast agent (GBCA) concentration-time curves used to extract VOF/VIFs from the SSS, the ICA, and the middle cerebral artery. Semi-quantitative parameters across each first-pass VOF/VIF were compared and the relationship between these parameters and GBCA dose was evaluated. Through a test–retest study in 12 patients, the repeatability of each semiquantitative VOF/VIF parameter was evaluated; and through comparison with histopathological data the accuracy of kinetic parameter estimates derived using each VOF/VIF and the extended Tofts model was also assessed. VOF_SSS_ provided a superior surrogate global input function compared to arteries, with greater contrast-to-noise (*p* < 0.001), higher peak (*p* < 0.001, repeated-measures ANOVA), and a greater sensitivity to interindividual plasma GBCA concentration. The repeatability of VOF_SSS_ derived semi-quantitative parameters was good to excellent (ICC = 0.717–0.888) outperforming arterial based approaches. In contrast to arterial VIFs, kinetic parameters obtained using a SSS derived VOF permitted detection of intertumoural differences in both microvessel surface area and cell density within resected tissue specimens. These results support the usage of an arrival-corrected VOF_SSS_ as a surrogate vascular input function for kinetic parameter mapping in brain DCE-MRI.

## Introduction

Dynamic contrast-enhanced (DCE) MRI has a developing role as an imaging tool for quantifying brain tumour microvasculature and tumour response to anti-angiogenic therapy^1–6^. In human clinical studies, non-invasive measurement of a suitable vascular input function (VIF) is essential for deriving microvascular kinetic parameters from brain DCE-MRI. Non-invasive measurement of a VIF with high temporal resolution is especially important when first-pass bolus tracking is necessary for quantitative kinetic analysis*,* such as when using the extended Tofts model^7–10^. Use of a fixed, experimentally derived population-averaged VIF for all subjects has been previously proposed^11–13^, thereby simplifying data acquisition, but large variations can occur in the actual VIF between subjects and scan visits due to both technical (e.g. differences in injection timing and dose) and patient specific factors (e.g. cardiac output, haematocrit, caffeine intake and atherosclerosis related vessel narrowing)^11,14,15^. For this reason methods have also been developed that enable simultaneous measurement of plasma gadolinium-based contrast agent (GBCA) concentration changes in both the blood and tissue under study, permitting VIF to be measured on an individual patient basis^11,14,16^.

In DCE-MRI studies of brain tumours the ideal choice for defining an individual VIF is the feeding artery of the tumour but due to either data acquisition constraints, the small size of the feeding vessel or lack of the feeding artery within the imaging field of view (FOV) this is often not possible^11,16–18^. Large intracranial arteries such as the internal carotid artery (ICA) and middle cerebral artery (MCA) are therefore often used as a surrogate global VIF measurement^11,16,19^. Use of the superior sagittal sinus (SSS), a venous structure, as a surrogate global input function has also been adopted, however, in numerous cross-sectional and longitudinal DCE-MRI studies^1,2,5,11,16,19–26^. The presented rationales for the use of the SSS vascular output function (VOF) as a surrogate global input function were said to be an assumption that the venous and arterial GBCA concentration is equal; that the degree of dispersion between arterial and venous structures in the brain during the first-pass circulation of the GBCA bolus is minimal; and that due to its larger comparative size the SSS is less susceptible to partial volume errors (PVE) than smaller intracranial arteries such as the ICA^8^.

The most commonly used 3D DCE-MRI acquisition is the 3D spoiled gradient echo method, and a VIF (plasma GBCA concentration-time curve) is typically determined from GBCA related magnitude changes in signal intensity^27^. Using a whole-brain, low-dose high temporal resolution (LDHT) axial 3D spoiled gradient-recalled echo sequence and a magnitude-based determination method we simultaneously measured a VOF/VIF from the SSS and large intracranial arteries (ICA, MCA) in a brain tumour patient cohort. Through this we sought to compare and understand features of each VOF/VIF and evaluate the respective ability of each VOF/VIF to accurately capture interindividual changes in patient dosing and plasma GBCA concentration. Through an included test–retest study we sought to establish the respective reproducibility of parameters derived from these arterial and venous VOF/VIFs; and through comparison with resected tumour specimens in a patient cohort, evaluate the ability of kinetic parameters derived using each VOF/VIF to detect intertumoural differences in histopathological data.

## Methods

### Study population

Previously acquired dual temporal resolution (DTR), dual injection DCE-MRI data in three groups of patients were analysed for this study: twenty-five patients with newly diagnosed WHO grade IV glioma synonym glioblastoma (GBM); twenty-nine patients with sporadic vestibular schwannoma (VS) listed for either radiological surveillance or treatment with surgery or stereotactic radiosurgery (SRS); and twelve patients with neurofibromatosis type 2 (NF2) related VS undergoing treatment with the anti-vascular endothelial growth factor (anti-VEGF) antibody, bevacizumab (Avastin ©). Ethical approvals were in place from the National Research Ethics Service Greater Manchester North-West research ethics committee (REC references: 13/NW/0131, 13/NW/0247 and 15/NW/0429). All patients had provided informed consent for study participation and later analysis of their MRI data and all research was performed in accordance with the Declaration of Helsinki and with local guidelines and policies.

### MR imaging

The 25 patients with GBM and 29 patients with sporadic VS were all imaged once on a 1.5 T scanner (Philips Achieva, Best, Netherlands). Thirteen of the included patients with GBM and twenty-two of the included patients with sporadic VS had been recruited and scanned as part of previous published studies at our institution^1,2,6^. The twelve patients with NF2-related VS had similarly been recruited as part of an earlier published study investigating bevacizumab (Avastin ©) related changes in DCE-MRI derived kinetic parameters in VS and these patients had been imaged twice at 1.5 T: pre-treatment (day 0) and 3 months (day 90) following bevacizumab (Avastin ©) treatment.

DCE-MRI data was acquired using a previously described DTR, dual injection technique^6,16,28^. Single dose macrocyclic GBCA (gadoterate meglumine; Dotarem, Guerbet S.A.) was used at a dose of 0.2 ml/kg. For VOF/VIF estimation and as the first part of this DTR technique, a low-dose fixed volume pre-bolus (either 2 or 3mls) of GBCA was administered over 1 s during acquisition of a high temporal resolution (LDHT) DCE-MRI dataset. All intravenous injections were performed using a two-cylinder power injector (MEDRAD® Spectris Solaris EP, Bayer, PA, US). The GBCA and 0.9% saline are contained within separate cylinders and the pre-bolus injection was followed by a chaser of 20 ml of 0.9% saline administered at the same rate (2 or 3 ml/s). A 3D spoiled gradient recalled echo (GRE) sequence with axial slab orientation and anterior–posterior frequency encoding was used for data acquisition and acquisition parameters for this LDHT acquisition were as follows: flip angle of 20°, TR/TE of 2.5 ms/0.696 ms, SENSE acceleration factor of 1.8, reconstructed matrix size of 96 × 96 × 22, voxel size of 2.5 × 2.5 × 6.35 mm^3^, pixel bandwidth of 700 Hz, frame duration (Δt) 1.0 s (n = 300)^6,16,28^. The minimum TE and fixed volume low GBCA dose used for the LDHT DCE series and VOF/VIF estimation was designed to avoid signal magnitude saturation and expected to produce minimal T2* and water exchange effects^29–31^.

As the second part of this DTR DCE-MRI technique, a full-dose of GBCA (dose = 0.2 ml/kg ·weight – dose of pre-bolus) was administered at the same rate (2 to 3 ml/s) as the pre-bolus (followed by a chaser of 20 ml of 0.9% saline administered at the same rate) during acquisition of a high-spatial resolution (FDHS) sequence^6,28^. FFT (Fast Fourier Transform) reconstruction in the z-direction was used for both the low-dose high temporal resolution (LDHT) and full-dose high spatial resolution (FDHS) acquisitions, doubling the number of slices^6^. Variable flip-angle (VFA; α = 2°, 8°, 15° and 20°) acquisitions were undertaken prior to both the LDHT and FDHS DCE-MRI series for baseline longitudinal relaxation rate (R1_0_) mapping, and the spatial resolution of each VFA acquisition series was chosen to match the LDHT and FDHS DCE series respectively^6^.

**Figure 1:**
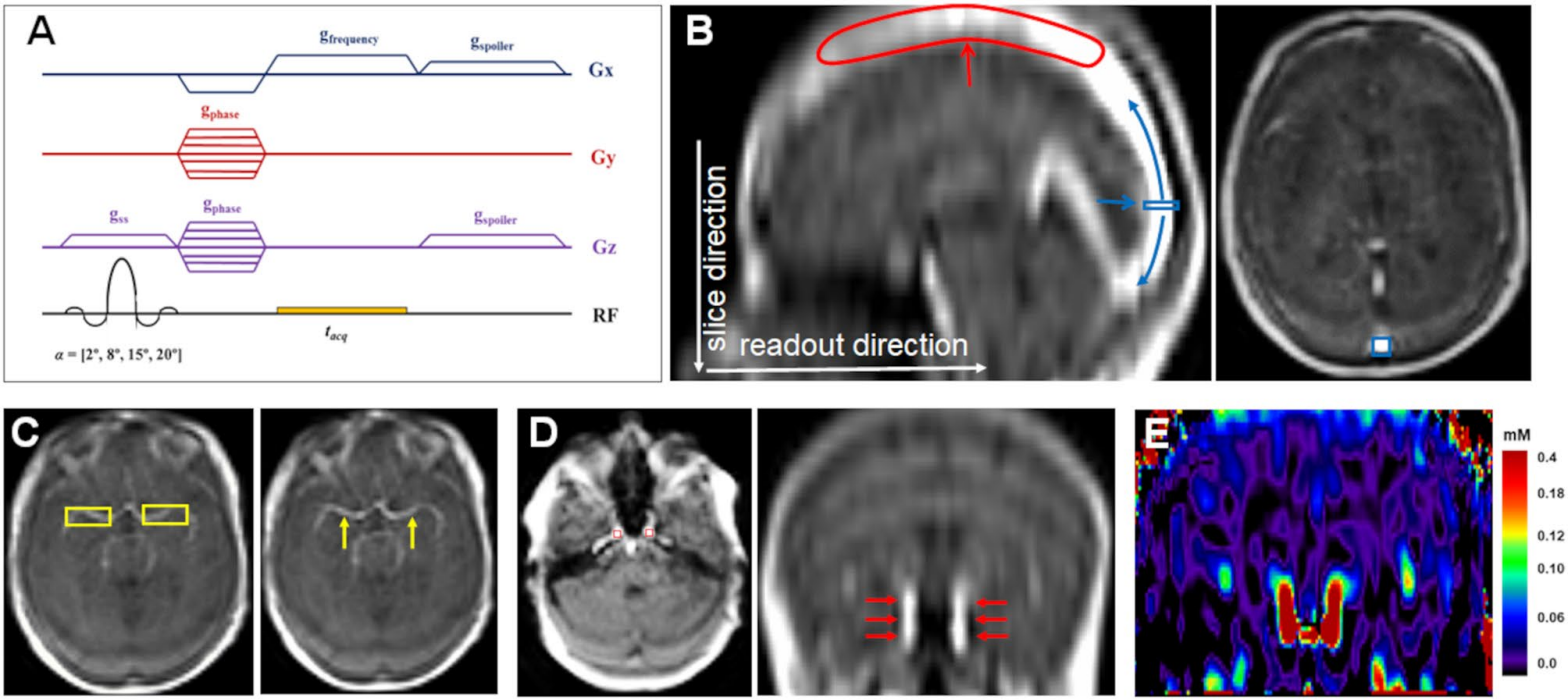
Magnitude-based input function extraction. (**A**): Pulse sequence diagram of 3D spoiled gradient recalled echo (GRE) sequence used for the variable flip angle (VFA) and low-dose high temporal resolution (LDHT) DCE-MRI acquisition, with axial slab orientation and anterior–posterior frequency encoding. Acquisition parameters: flip angle/s of 2, 8, 15, 20°; TR/TE of 2.5 ms/0.696 ms; SENSE acceleration factor of 1.8, reconstructed matrix size of 96 × 96 × 22, voxel size of 2.5 × 2.5 × 6.35 mm^3^, pixel bandwidth of 700 Hz, and frame duration (Δt) 1.0 s (n = 300). Pulse of frequency encoding/readout gradient (g_frequency_), phase encoding gradient (g_phase_) and slice select gradient (g_ss_) are shown along with signal acquisition time (t_acq_). Gradient spoilers (g_spoiler_) were applied along both the read and slice/slab selection directions and phase cycling with a phase increment angle of 117° was also used, allowing for more complete dephasing of residual transverse magnetization, and minimizing blood inflow-induced errors within each imaging slice. (**B**): Sagittal (*left panel*) and axial (*right panel*) signal-intensity (SI) images of a postcontrast time frame from the LDHT DCE-MRI series for a representative patient. Sagittal view shows that the anterior-middle portion of the SSS (red arrow) is included in the FOV of the 3D acquisition. This maximizes the number of RF pulses that the flowing blood experiences before it reaches the posterior portion of the SSS, helping to reduce blood inflow-induced errors. Following manual delineation of a small rectangle ROI within the vertical/posterior SSS (*blue arrow*), an automatic extraction method is used to identify voxels within neighbouring axial slices of the posterior SSS (*blue arrows*) that display maximum enhancement area under the SI curve within 30 s of the bolus arrival time (AUC_30_). A mean SI-time curve is then calculated from 20 voxels with the highest AUC_30_ and converted to a plasma GBCA concentration–time curve C_p_(t) using a literature value of blood R1_0_ of 0.694 s^-1^. (**C**): Axial SI images of a postcontrast LDHT DCE time frame for the patient shown in panel A showing the site of MCA VIF delineation. A pair of rectangle ROIs was drawn covering the horizontal segment of the MCA bilaterally (*yellow rectangles, left panel*). Using the described automatic method neighbouring contiguous axial slices were searched to identify twenty voxels within the MCA bilaterally (*yellow arrows, right panel*) that displayed maximum AUC_30_. (**D**): Axial (*left panel*) and coronal (*right panel*) SI images of a postcontrast LDHT DCE time frame for the patient shown in panel A showing the site of ICA VIF delineation. A pair of rectangle ROIs were manually drawn on an axial image section over the ICA (just distal to the carotid syphon) bilaterally. An automatic method was then used to search and identify twenty voxels within neighbouring contiguous axial slices of the carotid syphons (*red arrows, right panel*) that displayed maximum AUC_30_. (**E**): Coronal GBCA concentration C(t) image (mM) derived from the difference image ((ΔS = S(t)−S(0)) between the pre- and post-contrast SI images shown above. Whilst observed ring-like artefacts are present in the coronal SI images shown above, such artefacts are not present in either the 4D GBCA concentration—time images (where the VIF/VOF are measured) shown, or pharmacokinetic parameter maps derived from these 4D concentration–time images. GBCA = Gadolinium-based contrast agent; ICA = Internal carotid artery; MCA = Middle cerebral artery; SSS = Superior sagittal sinus.

To eliminate unsaturated flowing spins entering the imaging slab and improve the accuracy of VIF estimation^27,32^ a large 3D acquisition volume covering the top of the brain, the circle of Willis and the terminations of the internal carotid arteries bilaterally was used^16,33^. Throughout the FOV, the number of radiofrequency (RF) pulses and the gradient spoiling that spins in flowing blood received was also maximized through the use of a fast spoiled GRE sequence with short TR and phase cycling. Gradient spoilers were applied along both the read and slice/slab selection directions and phase cycling with a phase increment angle of 117° was used. This allowed for more complete dephasing of residual transverse magnetization, minimizing blood inflow-induced errors within each imaging slice^27,30,34^. A pulse sequence diagram of the 3D spoiled gradient recalled echo (GRE) sequence used for both the LDHT and FDHS acquisition is shown in Fig. 1A.

### Vascular input function extraction

For VOF/VIF extraction, acquired 4D LDHT DCE-MRI datasets (voxel size 2.5 × 2.5 × 6.35 mm^3^) were spatially aligned with and resliced to the FDHS data (voxel size 1 × 1 × 2 mm^3^) using SPM^35^. Although native LDHT datasets can also be used for input function extraction without prior co-registration and re-slicing, use of this co-registration step improved delineation of the blood vessel from surrounding tissues and permitted greater flexibility in ROI delineation and voxel selection. Plasma GBCA concentration-time curves, C_p_(t), were derived from signal intensity (SI)—time curves measured at three different sites: 1) vertical/posterior third of the SSS, VIF_SSS_; 2) the ICA just distal to the carotid syphon, VIF_ICA_: and 3) horizontal segment of the middle cerebral artery (MCA), VIF_MCA_. The first-pass data from these GBCA concentration-time curves were then fitted using a gamma variate function with a recirculation cut-off^36^, i.e.

$$ {\text{C}}_{{\text{p}}} \left( t \right) \, = Q\left( {t^{r} } \right)e^{ - (t/b)} ,{\text{ where Q}},{\text{ r and b are constants}}. $$

For each extracted VOF/VIF the following semi-quantitative parameters were derived and compared: the contrast-to-noise ratio (CNR), the bolus arrival time (BAT), the bolus peak-amplitude, the bolus peak width (FWHM, full-width at half-maximum) and the bolus peak∙FWHM product (PWP), a parameter associated with the area under the bolus curve.

For measurement of VOF in the SSS, blood inflow-induced errors were reduced by including the anterior and middle third of the SSS in the field of view thereby maximizing the number of RF pulses that the flowing blood experiences before it reaches the posterior third of the SSS. Following manual delineation of a small rectangle ROI within the vertical/posterior SSS (Fig. 1B), an automatic extraction method was used to search and identify voxels within this segment of the SSS that display maximum enhancement area under the SI curve within 30 s of the bolus arrival time (AUC_30_)^37^. Through this semi-automatic extraction method voxels with maximal AUC_30_ and thereby less inflow and PVE were chosen for inclusion in the VOF. The above semi‐automatic extraction method was also applied for VIF extraction from the ICA and MCA. A pair of rectangle ROIs was manually drawn on an axial image section over the ICA (just distal to the carotid syphon) or MCAs bilaterally respectively (Fig. 1). Similar to VOF_SSS_ an automatic method was then used to search and identify voxels within neighbouring contiguous axial slices that displayed maximum enhancement (AUC_30_).

For each VOF/VIF a mean SI-time curve was calculated from 20 voxels with the highest AUC_30_ and this mean SI-time curve was then converted to a plasma GBCA concentration-time curve C_p_(t) using previously described methods^33^. Due to the difficulties of accurately measuring the pre-contrast T1 of flowing blood using standard DCE-MRI sequences and the bias introduced through in vivo experiments^38–40^, a literature value of blood R1_0_ of 0.694 s^-1^ was used for the conversion^41,42^.

### Kinetic parameter analysis

To evaluate the effect of VIF approach on kinetic parameter estimates, high spatial resolution (1 × 1 × 2 mm^3^) voxelwise maps of the microvascular kinetic parameters K^trans^ (transfer constant), *v*_p_ (fractional plasma volume) and v_e_ (the fractional volume of extravascular extracellular space or EES) were derived using the extended Tofts model (ETM)^43^ and the previously described LEGATOS (LEvel and rescale the Gadolinium contrast concentrations curves of high-temporal TO high Spatial DCE-MRI) method^6^. Pre-surgery DCE-MRI datasets from 15 sporadic VS with available comparative tissue histology were chosen as test group for this analysis and for each VOF/VIF (SSS, ICA, MCA) separate kinetic parameter estimates were derived^43^. In addition to derivation of K^trans^, v_p_ and v_e_ within each tumour voxel through the ETM, the voxelwise intracellular fraction (v_i_) was also estimated through the relationship v_i_ = 1−v_e_−v_p._

The LEGATOS method for deriving high-spatial resolution kinetic parameter maps from DTR, dual-injection DCE-MRI data has been previously described^6^. In key step 1 of this method, errors through temporal jitter uncertainty are reduced through construction of a merged DTR 4D GBCA concentration volume containing a high temporal (HT) resolution ‘arterial’ phase followed by a later low temporal but high spatial (HS) resolution ‘parenchymal’ phase^6,44^. In key step II the high temporal but low spatial resolution arterial phase of each pixel concentration curve is then re-scaled using the LEGATOS method and a derived pixelwise calibration ratio, to increase the spatial resolution of derived kinetic parameter maps. For the LEGATOS method, a combined VOF/VIF is adopted. The whole C_p_ (*t*) from the LDHT-derived VOF/VIF is concatenated with the dose-calibrated late part of the C_p_ (*t*) measured from the FDHS-derived VOF/VIF, and is used for kinetic analysis of the LEGATOS-generated 4D high spatiotemporal resolution GBCA concentration volume^6^.

The BAT for each tissue voxel is calculated as part of each fitting procedure and the *C*_p_(t) measured from each VOF/VIF time-shifted and aligned with the BAT of each tissue voxel contrast agent concentration-time curve^6^. As part of the fitting procedure and to assess the discrepancy between the original data and the derived curve a map of scaled fitting error (SFE) was also generated, with voxels displaying an SFE value > 50% being excluded from the tumour statistics^6,45^. For all patients, the SFE and derived kinetic parameter maps, both before and after exclusion of voxels with SFE > 50%, were visually inspected to confirm the acceptance of using SFE > 50% for outlier tumour voxel exclusion^6^.

### Tissue analysis

For the 15 resected sporadic VS, previously obtained tissue metrics were compared against derived kinetic parameter estimates using each VOF/VIF^6^. Collected paraffin blocks from each case were cut into serial 5-µm tissue sections and assessed for cell density (haematoxylin and eosin, H&E), vascular permeability (fibrinogen) and microvessel surface area (CD31) using immunoperoxidase immunohistochemistry and established protocols^1,2,6^. Ethical approval was obtained for tissue analyses (REC reference 15/NW/0429 and 19/NS/0167) and detailed protocols are described in prior publications^1,2,6^.

### Statistical analysis

The SPSS statistical software package (version 25, IBM Corp.) and Stata version 11 were used for all statistical tests. Extracted semi-quantitative parameters were compared across each vessel (SSS, ICA and MCA) using a repeated-measures ANOVA with Greenhouse–Geisser correction for non-sphericity. Post hoc analysis of pairwise comparisons between different VOF/VIF locations was performed using the Bonferroni method. Due to the design of a fixed volume injection approach, the dose of pre-bolus slightly varied with the patient body mass, whilst keeping the same length of the bolus (1 s) for all subjects. This allowed the sensitivity of different VOF/VIFs to small variations in GBCA dose to be assessed. For each vessel region (SSS, ICA, MCA) the relationship between extracted features and administered GBCA dose (mmol/kg) was assessed using scatterplots and correlation analysis. Correlation analysis was also used to evaluate the relationship of the GBCA bolus arrival time delay between each arterial (ICA, MCA) VIF and VOF_SSS_, and differences in bolus peak-amplitude, bolus peak width (FWHM) and the bolus PWP between the SSS and either ICA or MCA. In particular, the correlation of the BAT delay with either the absolute difference in each semiquantitative parameter (bolus peak-amplitude, bolus peak FWHM, bolus PWP) or the ratio of each parameter between the SSS and ICA/MCA (e.g., Peak_SSS_/Peak_ICA/MCA_ ratio) was assessed.

The intra-subject repeatability of each VOF/VIF semiquantitative parameter was assessed across the twelve patients with NF2 related VS who were imaged twice using the test–retest coefficient of variation (CoV). The CoV is the standard deviation, **σ**, across all measurements for each subject, divided by the mean, **μ**, for that subject. For a group of **N** subjects the global test–retest CoV is defined as $\sqrt{\sum {(\sigma /\mu )}^{2}/N}$^46,47^. As a supporting measure of repeatability the average measures intraclass correlation coefficient (ICC) of each VOF/VIF semiquantitative parameter across the two visits was also calculated using an absolute-agreement, 2-way mixed-effects model^48^. The inter-tumour correlation between DCE-MRI derived parameter estimates (*K*^trans^, *v*_p_, *v*_e_ and v_i_) and tissue-derived metrics (H&E cell density, CD31% microvessel surface area, fibrinogen optical density) for the 15 resected sporadic VS are reported as Pearson’s product moment correlation coefficient (*r*).

## Results

### Compared to large intracranial arteries (ICA, MCA) VOF_SSS_ provides a global surrogate input function with higher CNR, higher peak and higher peak∙FWHM product (PWP)

**Figure 2:**
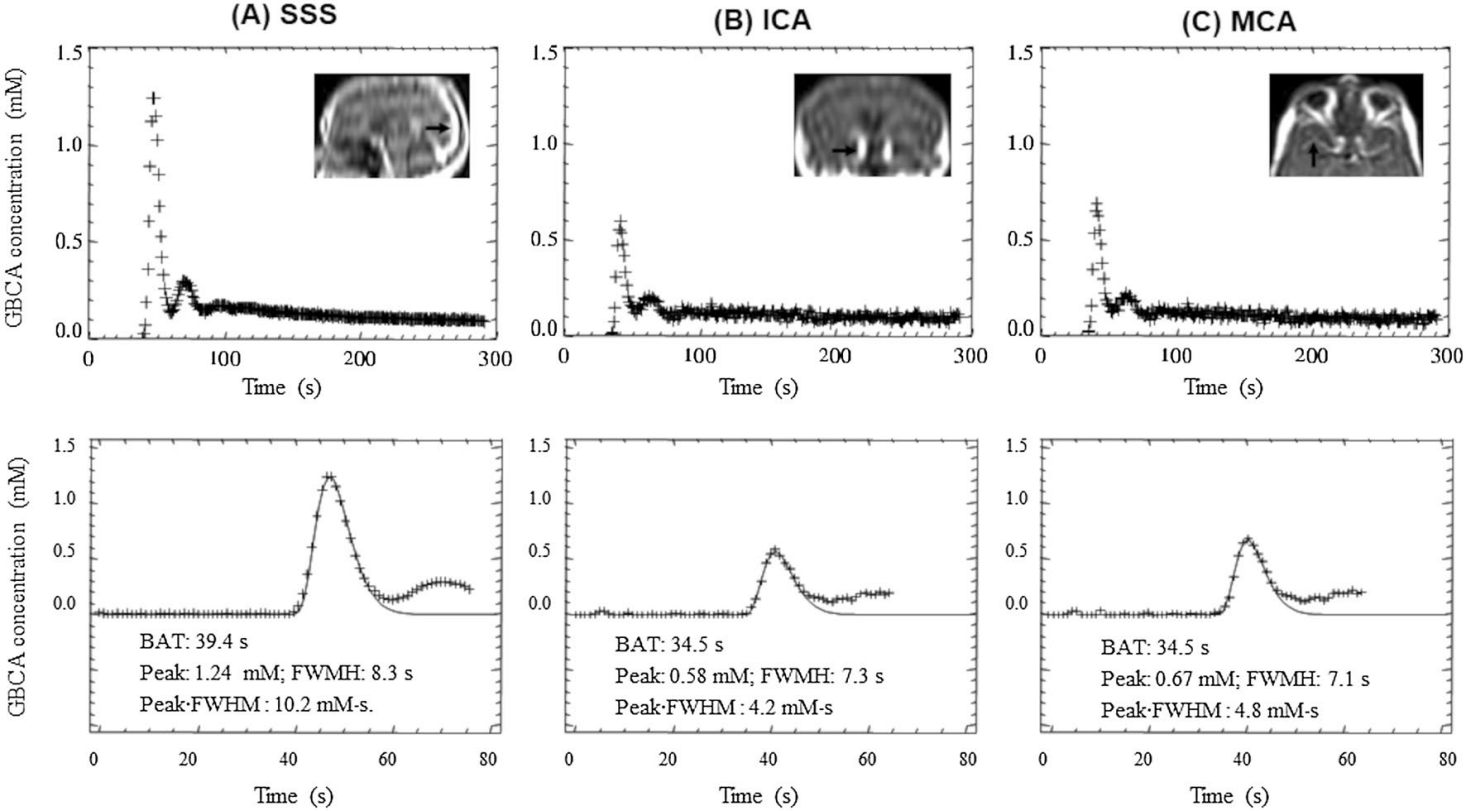
Typical plasma GBCA concentration–time curves *C*_p_(t) (*top row*) derived from the vertical segment of the SSS (**A**); the ICA (**B**); and horizontal segment of the middle cerebral artery MCA (**C**) following a bolus injection of 2mls of GBCA (0.016 mmol/kg) at a rate of 2 ml/s. The first-pass data are fitted using a gamma variate function, which excludes contrast-agent bolus recirculation (*bottom row*). Images obtained from a patient with a sporadic VS imaged at 1.5 T. GBCA = Gadolinium-based contrast agent; ICA = Internal carotid artery; MCA = Middle cerebral artery; SSS = Superior sagittal sinus.

In Fig. 2, representative plasma GBCA concentration curves *C*_p_(t) derived from the SSS, the ICA, and the horizontal segment of the middle cerebral artery MCA following a bolus injection of 0.016 mmol/kg of GBCA are shown. As shown in Table 1, across all patient datasets the CNR of VOF_SSS_ (mean CNR = 197.2 ± 95.7) was significantly higher than either VIF_ICA_ (mean CNR = 51.3 ± 25.9, *p* < 0.001) or VIF_MCA_ (52.9 ± 22.7, *p* < 0.001, repeated measures ANOVA). There was no difference in CNR between VIF_ICA_ and VIF_MCA_ (*p* > 0.05, repeated measures ANOVA, Table 1). Compared to either the VIF_ICA_ or VIF_MCA,_ VOF_SSS_ displayed significantly longer BAT (*p* < 0.001), higher peak (*p* < 0.001), larger bolus PWP (*p* < 0.001) and a non-significantly narrower FWHM (*p* > 0.05, repeated measures ANOVA, Table 1). There was no significant difference in either BAT (*p* > 0.05) or FWHM (*p* > 0.05) between VIF_ICA_ and VIF_MCA,_ but VIF_MCA_ displayed a higher peak (*p* < 0.001) and higher PWP (*p* < 0.001) than VIF_ICA._

**Table 1:** Comparison of semi-quantitative VOF/VIF features extracted from superior sagittal sinus (SSS), internal carotid artery syphon (ICA) and middle cerebral artery (MCA).

VOF/VIF Feature (n = 66)	Mean (± S.D)			*p* value		
	SSS	ICA	MCA	SSS & ICA	SSS & MCA	ICA &MCA
Contrast-to-noise ratio (CNR)	197.2 (95.7)	51.3 (25.9)	52.9 (22.7)	**< 0.001**	**< 0.001**	0.99
Bolus arrival time (BAT, seconds)	37.9 (2.43)	33.5 (2.41)	33.5 (2.39)	**< 0.001**	**< 0.001**	0.99
Peak amplitude (mM)	0.99 (0.30)	0.58 (0.19)	0.73 (0.29)	**< 0.001**	**< 0.001**	**< 0.001**
Full-width at half-maximum of bolus peak (FWHM, s)	9.86 (2.36)	10.2 (3.44)	10.3 (3.52)	0.45	0.33	0.99
Bolus peak∙FWHM product (PWP, mM⋅s)	9.56 (3.19)	5.80 (2.39)	7.19 (3.05)	**< 0.001**	**< 0.001**	**< 0.001**

Significant values are in [bold].

Mean (± S.D) of each semi-quantitative parameter shown. Extracted semi-quantitative parameters were compared using a repeated-measures ANOVA with Greenhouse–Geisser correction for non-sphericity. Post hoc analysis of pairwise comparisons between different timepoints was performed using the Bonferroni method. *P* value shows comparison between each VIF location (SSS, ICA, MCA).

*BAT* Bolus arrival time, *ICA* Internal carotid artery, *MCA* Middle cerebral artery, *PWP* Bolus peak∙FWHM product, *SSS* Superior sagittal sinus, *VIF* Vascular input function, *VOF* Vascular output function.

**Figure 3:**
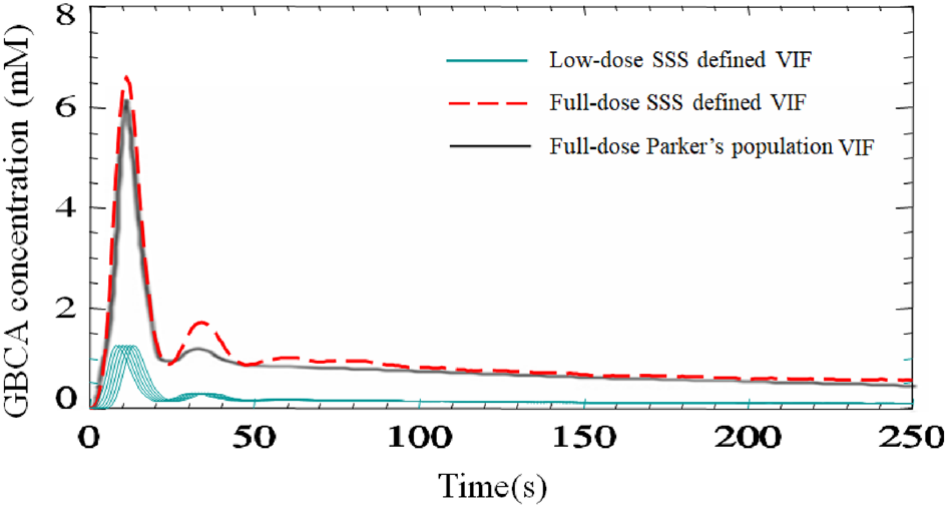
Comparison between an individual patient derived vascular output function from the SSS (VOF_SSS_) and the population VIF measured in the descending aorta by Parker et al. VOF extracted from the vertical segment of the superior sagittal sinus (SSS) in the same patient as shown in Fig. 2. Six low-dose (0.016 mmol/kg of GBCA) VIFs (*blue solid curves*) derived from the SSS were time-shifted and summed to generate a full-dose (0.1 mmol/kg) input function (*red dashed curve)*. The shape and amplitude of this summed SSS derived VIF shows high similarity with Parker's full-dose VIF (*black solid curve*), in keeping with the observed lack of bolus widening (bolus FWHM) between the large arteries and the SSS.

In Fig. 3 a comparison is shown between an individual patient derived VOF_SSS_ and the population VIF measured in the descending aorta by Parker et al^13^. In keeping with the observed lack of bolus widening (bolus FWHM) between the large intracranial arteries and the SSS, after converting the patient derived VOF_SSS_ to a full-dose input function by summing several time-shifted low-dose GBCA concentration–time curves^49^, there was a close resemblance between the summed SSS defined VOF and the Parker population VIF^13^.

### VOF_SSS_ demonstrates a greater sensitivity to interindividual changes in plasma GBCA concentration compared to arterial approaches

**Figure 4:**
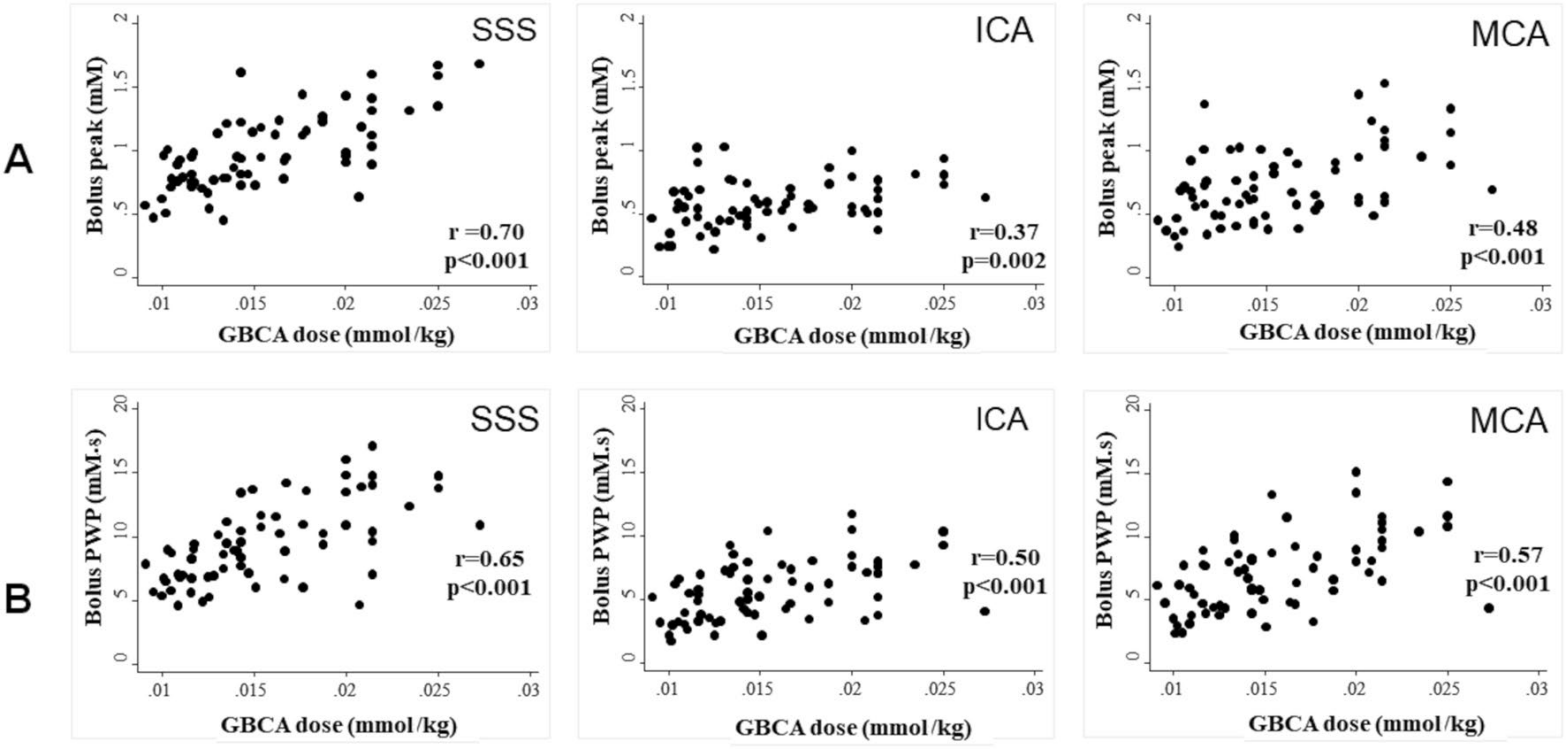
Relationship between extracted VOF/VIF features and administered GBCA dose for VOF_SSS_, VIF_MCA_ and VIF_ICA_. Top row: Scatter plots of bolus-peak (mM) vs administered GBCA dose (mmol/kg). Bottom row: Scatter plots of GBCA bolus peak∙FWHM product (PWP, mM⋅s) vs administered GBCA dose. Pearson’s product moment correlation coefficient (r) shown with associated p value. FWHM = full-width at half-maximum; ICA = Internal carotid artery; MCA = Middle cerebral artery; PWP = bolus peak∙FWHM product; SSS = Superior sagittal sinus.

Across all sixty-six patients, body weight adjusted GBCA dose for the pre-bolus injection and LDHT acquisition varied from 0.0091 to 0.027 mmol/kg with a mean injected dose of 0.016 mmol/kg. As shown in Fig. 4 both bolus peak (mM) and bolus PWP (mM**⋅**s) correlated significantly (*p* ≤ 0.002) with body weight adjusted GBCA dose with the strongest correlation observed for VOF_SSS_ peak (r = 0.70, *p* < 0.001) and VOF_SSS_ bolus PWP (r = 0.65, *p* < 0.001). No significant correlation was observed between body weight adjusted GBCA dose and bolus peak width (FWHM) for any derived VOF/VIF (*p* > 0.05, Pearson’s correlation co-efficient). As shown in Table 2, no significant correlation (*p* > 0.05) was observed between GBCA bolus PWP ratio (PWP_SSS_/PWP_ICA_ or PWP_SSS_/PWP_MCA_) and the BAT difference between SSS and ICA or between SSS and MCA respectively. A longer time interval between BAT_ICA_ and BAT_SSS_ correlated with an increase in the FWHM_SSS_/FWHM_ICA_ ratio (r = 0.35, *p* = 0.004) and was non-significantly associated with a lower Peak_SSS_/Peak_ICA_ ratio (r = − 0.18, *p* = 0.15).

**Table 2:** Relationship between the GBCA bolus arrival time delay and differences in semiquantitative parameters between the arterial (ICA, MCA) VIFs and venous (SSS) VOF.

VOF/VIF Feature (n = 66)	BAT difference between SSS and ICA (seconds)	BAT difference between SSS and MCA (seconds)
Difference in bolus peak (mM) between SSS and ICA/MCA	r = **− **0.17 p = 0.16	**r = − 0.30** **p = 0.02**
Difference in bolus FWHM (seconds) between SSS and ICA/MCA	**r = 0.35** **p = 0.004**	**r = 0.33** **p = 0.007**
Difference in bolus PWP (mM⋅s) between SSS and ICA/MCA	r = 0.15 p = 0.22	r = **− **0.05 p = 0.74
Peak_SSS_/Peak_ICA or MCA_ ratio	r = **− **0.18 p = 0.15	r = **− 0.28** **p = 0.03**
FWHM_SSS_/FWHM_ICA or MCA_ ratio	**r = 0.35** **p = 0.004**	**r = 0.36** **p = 0.003**
PWP_SSS_/PWP_ICA or MCA_ ratio	r = **− **0.01 p = 0.96	r = **− **0.12 p = 0.34

Significant values are in [bold].

Pearson’s product moment correlation coefficient (r) shown with associated p value.

*BAT* Bolus arrival time, *ICA* Internal carotid artery, *MCA* Middle cerebral artery, *PWP* Bolus peak∙FWHM product, *SSS* Superior sagittal sinus, *VIF* Vascular input function, *VOF* Vascular output function.

### VOF_SSS_ demonstrates greater repeatability compared to arterial VIF approaches

Table 3 shows the intra-subject variability and repeatability of each semiquantitative VOF/VIF parameter across the twelve patients with NF2-related VS. In the case of VOF_SSS,_ global CoV values for BAT, bolus peak, bolus FWHM and bolus PWP were 3.98%, 17.0%, 16.8% and 12.4% respectively. Except for BAT, global CoV values for VIF_SSS_ were lower than the corresponding global CoV values for VIF_ICA_ or VIF_MCA._ Across all semi-quantitative parameters extracted from the VOF_SSS_ repeatability was good to excellent (ICC = 0.717–0.888)^50,51^.

**Table 3:** Repeatability of semi-quantitative parameters extracted from VOF_SSS_ and arterial (ICA, MCA) VIFs.

Semi-quantitative parameter		Global CoV	Mean CoV ± SD	ICC^a^
BAT	SSS	3.98	3.10 (2.60)	**0.794**
	ICA	3.18	2.70 (1.76)	**0.886**
	MCA	3.78	3.29 (1.93)	**0.809**
Peak	SSS	17.0	11.4 (13.2)	**0.857**
	ICA	23.0	17.6 (15.4)	**0.778**
	MCA	24.2	19.9 (14.5)	**0.621**
FWHM	SSS	16.8	13.7 (10.2)	**0.717**
	ICA	21.5	19.4 (9.78)	**0.552**
	MCA	24.2	21.7 (11.2)	**0.09**
PWP	SSS	12.4	9.98 (7.72)	**0.888**
	ICA	16.1	14.5 (7.40)	**0.909**
	MCA	20.9	17.3 (12.2)	**0.794**

Significant values are in [bold].

Data shown from 12 patients with NF2-related VS imaged pre-treatment (day 0) and 3 months (day 90) following treatment with bevacizumab (Avastin ©). Individual patient level coefficient of variation (CoV) values reported alongside mean (+ /− S.D) and global CoV.

^a^Average measures ICC estimates are reported based on an absolute-agreement, 2-way mixed-effects model.

*BAT* Bolus arrival time, *ICA* Internal carotid artery, *ICC* Average measures intraclass correlation coefficient, *MCA* Middle cerebral artery, *PWP* Bolus peak∙FWHM product, *SSS* Superior sagittal sinus, *VIF* Vascular input function, *VOF* Vascular output function.

### Kinetic parameters obtained using a SSS derived VOF permitted detection of intertumoural differences in histopathological data

**Figure 5:**
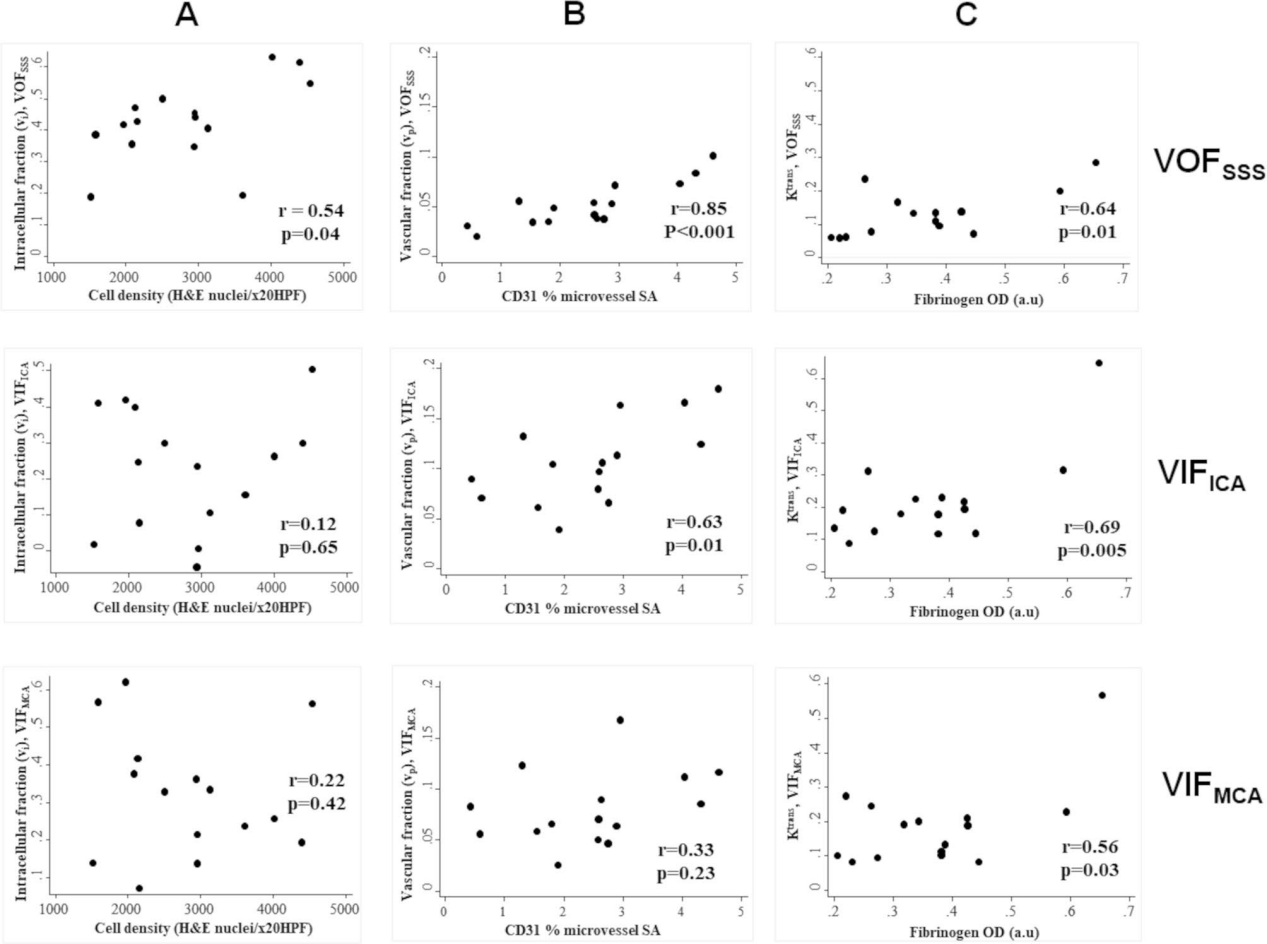
Scatterplot comparison of histopathological data with kinetic parameter estimates (v_i_, v_p_, K^trans^) derived using different VOF/VIF approaches. (**A**): Intertumour scatterplot comparison of mean tumour intracellular fraction (*v*_*i*_, no units) estimates against mean H&E cell density (nuclei/ x20HPF). *v*_*i*_ estimates derived using VOF_SSS_ (*top row*), VIF_ICA_ (*middle row*) and VIF_MCA_ (*bottom row*) shown. (**B**) Intertumour scatterplot analysis of mean vascular fraction (*v*_p,_ no units) against mean CD31% microvessel surface area (SA). *v*_*p*_ estimates derived using VOF_SSS_ (*top row*), VIF_ICA_ (*middle row*) and VIF_MCA_ (*bottom row*) shown. (**C**) Intertumour scatterplot analysis of mean tumour *K*^trans^ (min^-1^) against mean fibrinogen optical density (OD). K^trans^ estimates derived using VOF_SSS_ (*top row*), VIF_ICA_ (*middle row*) and VIF_MCA_ (*bottom row*) shown.

**Figure 6:**
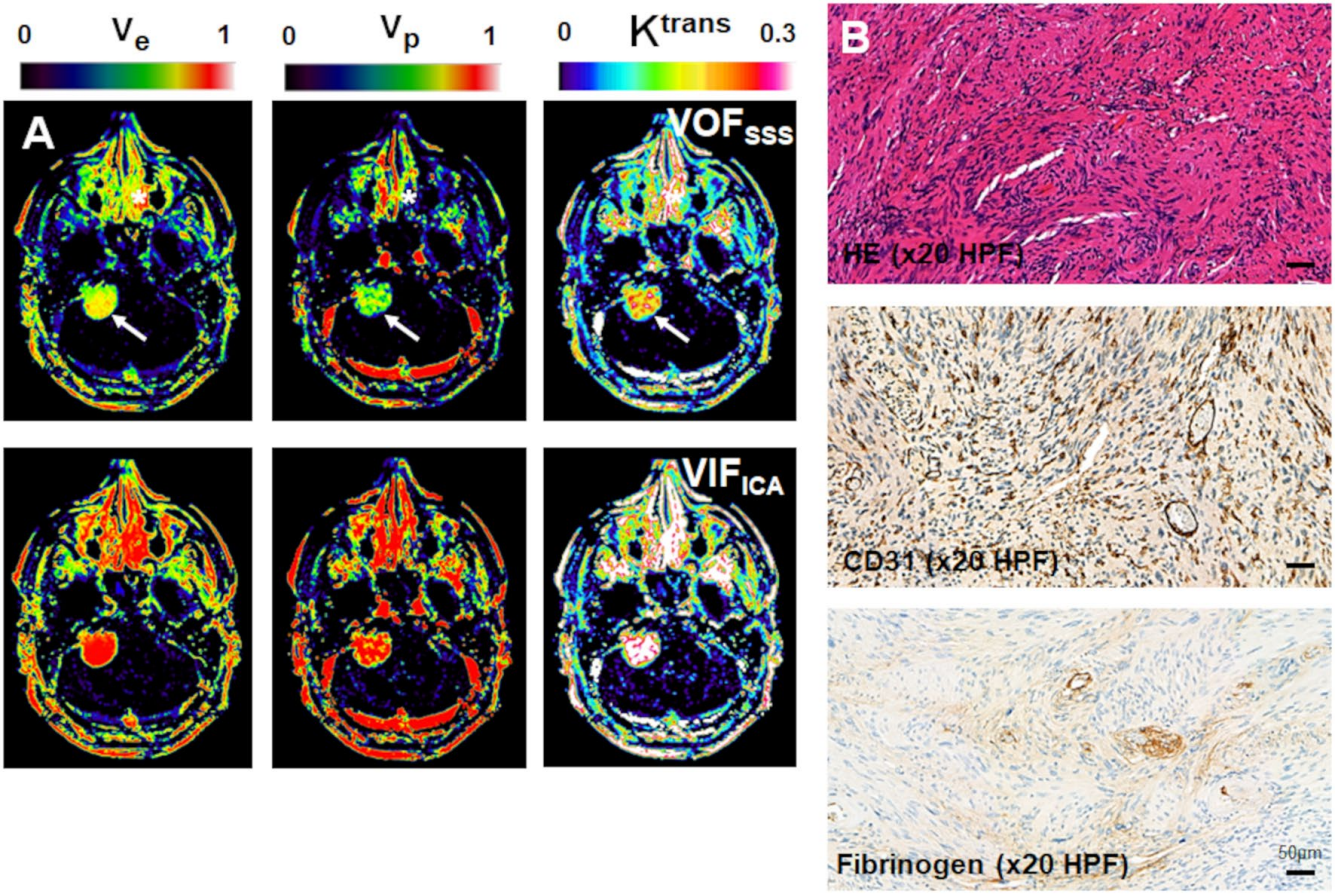
Parameter maps and tissue sections from a large sporadic VS demonstrates overestimation of kinetic parameters (v_e_, v_p_, K^trans^) with use of VIF_ICA_. (**A**): Representative LEGATOS derived kinetic parameter maps from a patient with a large right sided sporadic VS. From left to right: parametric *v*_e_ map; parametric *v*_p_ map and parametric *K*^trans^ map. Note the comparatively higher v_e_ v_p_ and K^trans^ estimates within the tumour (*arrow*) and nasal mucosa (*) when using the ICA derived vascular input function (VIF_ICA_). (**B**): Representative tissue sections from the tumour shown in panel A. From top: Haematoxylin and eosin-stained section (HE-x20HPF); CD31 immunostain for microvessels (brown; immunoperoxidase –x20HPF); fibrinogen immunostain for perivascular leak (brown; immunoperoxidase –x20HPF). In keeping with the VOF_SSS_ kinetic parameter estimates, the tumour is moderately cellular (mean tumour v_i_ ~ 0.35) with some regions of moderate/high microvessel density (CD31) and perivascular leak (fibrinogen). The VIF_ICA_ and VIF_MCA_ kinetic parameter estimates, however, overestimate both the size of the tumour extravascular-extracellular space (v_e_) and the degree of tumour vascularity (v_p_).

In Fig. 5 the inter-tumour correlation between LEGATOS derived kinetic parameter estimates and tissue metrics for each VOF/VIF are shown. There was a significant correlation between cell density and mean tumour *v*_i_ when using VOF_SSS_ (r = 0.54, *p* = 0.04, Fig. 5A). No such correlation was seen, however, when using either VIF_ICA_ or VIF_MCA_. In many tumours there was overestimation of v_e_ when using arterial VIF (6/15 and 4/15 VS had v_e_ > 0.7 when using VIF_ICA_ and VIF_MCA_ respectively), and such high EES fractions were not evident on collected tissue from these tumours (Fig. 6). For VOF_SSS_ and VIF_ICA_ a significant positive correlation was seen between CD31% microvessel surface area and mean tumour *v*_p_ (*p* < 0.05), but this correlation was strongest for v_p_ maps derived using VOF_SSS_ (r = 0.85, *p* < 0.001, Fig. 5B). Estimates of both v_p_ and K^trans^ when using an arterial VIF were higher than VOF_SSS_ derived estimates. Across all VOF/VIF approaches, there was a correlation of K^trans^ with both CD31% microvessel surface area (*p* < 0.05) and perivascular leak as measured through fibrinogen optical density (*p* < 0.05).

## Discussion

Compared to large intracranial arteries (ICA, MCA), an arrival-corrected VOF derived from the SSS (VOF_SSS_) can provide a superior surrogate global input function for kinetic parameter analysis in brain DCE-MRI, demonstrating higher CNR, higher peak and a greater sensitivity to interindividual changes in plasma GBCA concentration. Through an included test–retest study we demonstrate that semi-quantitative parameters derived from VOF_SSS_ display greater repeatability than arterial based approaches. Furthermore, through comparison with matched tissue datasets in patients with resected sporadic VS we demonstrate that microvascular kinetic parameters obtained using an arrival-corrected VOF_SSS_ permit evaluation of intertumoural differences in microvessel surface area and cell density, a feature not seen with large artery based VIFs.

Previous studies comparing arterial and venous based VOF/VIFs have reported similar results to our findings, and several factors can be hypothesized to contribute to the observed higher CNR and higher peak measured within VOF_SSS_ compared to large arteries^19,32^. Keil et al. demonstrated that arterial VIF showed lower peak C_p_ values and more often led to implausibly high v_e_ values in subsequent pharmacokinetic parameter fitting compared to a SSS derived VOF^19^. The larger cross-sectional area of the SSS relative to both the ICA and MCA may serve to reduce PVE and contribute to the higher CNR, higher peak GBCA bolus measured^32,39^. Reduced ‘in-flow’ effects secondary to lower flow velocities within the SSS may also contribute to the higher CNR observed^25,52–54^. To maximise T1 weighting, a 3D T1W GRE sequence with the shortest TE was used for the DCE-MRI acquisition. Such GRE sequences are, however, prone to ‘in-flow’ related enhancement, which can lead to a significant attenuation of contrast enhancement by GBCA and subsequent reduction of measured VIF CNR^27,30^. Within our study, measures were taken to prevent these ‘in-flow’ artefacts such as RF phase cycling, use of a large acquisition volume covering the whole brain, and orientation of the read gradient of the 3D slab parallel to the vessels. It is, however, possible that such ‘in-flow’ related artefacts still occurred^39^ and because the contrast between flowing and static tissue depends directly upon the flow velocity^53,54^, the higher velocity arteries are much more prone to increased ‘in-flow’ effects and resulting lower CNR than lower velocity venous structures such as the SSS^25,39,52^.

An additional factor that may have contributed to the observed decreased CNR and peak within arterial VIFs relative to the SSS is differences in inter- and intravoxel velocity dispersion within these vessels^39^. Compared to large intracranial arteries, blood flow within the SSS is thought to be less turbulent and more laminar, with a parabolic velocity profile^55,56^. Assuming laminar flow and a parabolic velocity profile, then the velocity v of a given proton spin across a circular vessel, is a function of distance to the isocentre of the flow, i.e., *v(r)* = *v*_max_ (1−*r*^2^/*a*^2^), where *a* is the radius of the vessel, *r* is the distance between the position of the flowing spin and the centre of the vessel, *v(r)* is the velocity at *r*, and *v*_max_ is the maximum velocity at the centre of the vessel. Whereas stationary spins are completely rephased during data acquisition, moving spins retain a degree of phase shift that is a function of both the first moment of the gradient M1 and the flow velocity *v*. The phase increases linearly with the flow velocity but there is no effect on the spatial location of the signal by 2D Fourier transform reconstruction because the velocity is set constant from view to view. The location would, however, be affected if acceleration in the vessel is heterogenous and in vessels where there is significant intra- and intervoxel velocity heterogeneity, phase dispersion and loss of CNR can occur. Moreover, changes of the parabolic velocity profile can influence the width of first-pass of the contrast bolus. In vivo blood flow measurement is considerably more complex, blood is non-Newtonian for example and the flow profile depends on the geometry of the vessels and the sampling point of the pulsed flow, i.e., the time points in the cardiac cycle. The field gradients for spatial encoding and the spoiler gradients used in our acquisition were not specifically designed for ‘flow compensation’ and extra spin dephasing caused by spins moving during the actual signal encoding may have occurred. Such flow-induced loss of phase coherence increases with higher velocity blood flow and may in addition to PVE and residual ‘in-flow’ effects explain the lower CNR and lower peak measured within arterial VIFs compared to VOF_SSS._

Within this study a rapid bolus injection protocol was used, with a compact low-dose bolus of GBCA injected over 1 s. Following injection into an upper limb vein, the GBCA bolus is expected to undergo a degree of dispersion and widening as it passes through first the pulmonary and then cerebral circulation. This dispersion process is commonly modelled as a mathematical convolution with the vascular system of the brain and a simple yet useful model for the dispersion process might contain a single artery that feeds a set of parallel pathways (an arteriole, a blood-tissue exchange unit, and a venule) that drain into a single vein^57–61^. Data from our study, however, suggests that when first-pass low-dose GBCA bolus uptake curves are measured within the distal SSS such dispersion is minimal, with no significant difference in peak width (FWHM) between VOF_SSS_ and the VIF measured from feeding larger arteries such as the ICA and MCA**.** This absence of significance bolus dispersion between the ICA and SSS has also been reported in other studies and several factors may underlie this unexpected observation^8,39^. A large contributing factor is likely inaccuracies in the measurement of the VIF within the smaller arterial vessels due to increased PVE, increased vessel inflow effects and increased velocity dispersion of flowing blood relative to the SSS. Such effects likely contribute to the increased CNR seen within the VOF_SSS_ but also counterbalance the broadening effect by dispersion^8^.

Previous works have demonstrated that incorrect estimation of VIF peak and the bolus arrival time can have significant impact on pharmacokinetic parameter fitting and accuracy^62–65^. In particular the bolus arrival time in the SSS is 4–5 s later than the true input arrival time and a correction must be made for the delayed arrival of the contrast agent within the venous system by incorporating voxel-wise BAT estimation in DCE-MRI analysis^66,67^. Within this study use of a DTR acquisition incorporating high temporal sampling during pre-bolus injection was adopted, improving voxel-by-voxel estimation of BAT delay, and reducing fitting errors induced by uncertainty in time alignment of the VOF/VIF and tissue uptake curves. Alongside measurement effects, physiological factors may also play a role in increasing CNR and reducing the observed dispersion seen within the SSS relative to arterial VIF sources. The SSS serves as the principal draining vein for the cerebral cortex and it’s comparatively straight course, absence of valves, and wide diameter relative to large intracranial arteries means that laminar blood flow characteristics within it resemble that of similar sized arteries^22,24,25,68^. Indeed a similarity between the measured VOF within the SSS and Parker’s population averaged VIF has been reported in both this and other studies^13,19^. Human histological studies and microsphere studies in rhesus monkeys have demonstrated that within normal brain under physiological conditions, a small percentage of blood from the arteries passes through small pial arteriovenous shunts (12.5 µm or more in diameter) directly into cerebral veins and the SSS^69–71^ Direct shunting of blood into cerebral veins has also been demonstrated angiographically within supratentorial GBM and VS, although in the latter case the source of principal venous drainage is the transverse and sigmoid sinuses rather than the SSS itself^72–78^. Although in theory the presence of such shunts may serve to reduce dispersion of the GBCA bolus within the venous system, the predicted low volume of blood passing into the SSS via these direct channels makes them unlikely to be a significant factor in driving the observed VOF_SSS_ characteristics.

The presented study is one of the largest comparisons of different arterial and venous based input functions for brain DCE-MRI and to our knowledge one of the first studies to evaluate the sensitivity of different VOF/VIF approaches to interindividual differences in plasma GBCA concentration and histopathological data. A limitation of the present study though is that characterization of plasma GBCA concentration curves, C_p_(t), was limited to semi-quantitative parameters such as the bolus peak-amplitude, bolus width and GBCA bolus PWP. Future studies should seek to undertake more detailed and sophisticated shape analysis of the whole length of the VOF/VIF. In this study, a semi-automatic extraction method was employed to measure each VOF/VIF, in which the twenty vessel voxels with the highest AUC_30_ are selected and averaged together to create the final input curve ^18,37,79^. This is under the assumption that these voxels demonstrate less inflow effects and PVE. Although it is possible that such an approach may overestimate the true value of VOF_SSS,_ our demonstration that the VOF_SSS_ showed an initial peak height and first-pass bolus shape very close to the ‘gold standard’ Parker VIF model^13,80,81^, and that the variation in the VOF_SSS_ bolus peak was strongly correlated with dose variation, suggests that such overestimation was minimal and that peak estimates are not dominated by bolus shape distortion. Our demonstration that kinetic parameter estimates, obtained using VOF_SSS_ correlated well with tissue derived measures of microvessel surface area and cell density, in contrast to arterial VIFs that showed lower peak values and overestimation of v_e_, further supports the robustness of input function surrogate measurements from the SSS and the absence of significant peak overestimation through this semi-automatic voxel selection method. Larger studies incorporating matched imaging-tissue cohorts in a range of different tumours should, however, be undertaken to better evaluate the effect of VOF/VIF location and voxel extraction method on kinetic parameter accuracy.

## Conclusion

Accurate derivation of a vascular input function (VIF) is essential for quantitative kinetic analysis of brain DCE-MRI data. In this in vivo patient study, we compared VIFs extracted from either the internal carotid artery and its branches with an arrival-corrected vascular output function derived from the superior sagittal sinus (VOF_SSS_). We demonstrated that compared to large intracranial arteries VOF_SSS_ can provide a superior surrogate global VIF, with lower noise, higher repeatability, and greater sensitivity to interindividual changes in plasma GBCA concentration. Through comparison with matched histopathological data, we furthermore demonstrate that microvascular parameters obtained using a SSS derived VOF permitted evaluation of intertumoural differences in microvessel surface area and cell density. These results support the use of venous sinus-based approaches for input function extraction and pharmacokinetic parameter mapping in brain DCE-MRI.

## Data Availability

The datasets generated during and/or analysed during the current study are available from the corresponding author on reasonable request.

## Abbreviations

ANOVA: Analysis of variance
AUC30: Area under the enhancing curve within 30 s of the bolus arrival time, mM·s
BAT: Bolus arrival time, seconds (s)
Cp(t): Plasma GBCA concentration time course, mM
CD31: Cluster of differentiation 31 synonym platelet endothelial cell adhesion molecule (PECAM-1), an endothelial marker
CNR: Contrast to noise ratio
COV: Test–retest coefficient of variation, %
Δt: Update time/frame rate of DCE-MRI acquisition, seconds (s)
DCE-MRI: Dynamic contrast enhanced MRI
DTR: Dual temporal resolution
EES: Extravascular-extracellular space
ETM: Extended Tofts model
FDHS DCE: Full dose high spatial DCE
FOV: Field of view
FWHM: Full-width at half maximum of bolus curve or bolus width, seconds (s)
GBCA: Gadolinium-based contrast agent/s
GBM: Glioblastoma multiforme synonym WHO grade IV glioma
GRE: Gradient recalled echo
ICA: Internal carotid artery
ICC: Average measures intraclass correlation coefficient
K^trans^: Volume transfer constant, min^-1^
LDHT DCE: Low-dose high temporal DCE
LEGATOS: LEvel and rescale the GAdolinium contrast concentrations curves of high temporal TO high spatial
MCA: Middle cerebral artery
NF2: Neurofibromatosis type II
PWP: Bolus peak∙FWHM product, a parameter associated with the area under the bolus curve, mM·s
PVE: Partial volume errors
R10: Native longitudinal relaxation rate (1/T10), s^-1^
ROI: Region of interest
SI: Signal intensity
SFE: Scaled fitting error, %
SSS: Superior sagittal sinus
T1W: Longitudinal relaxation (T1) weighted (MR imaging)
TE/TR: Echo time/ Repetition time, ms
v_e_: Volume of extravascular-extracellular space per unit volume of tissue, %
v_i_: Volume of intracellular space per unit volume of tissue (estimated), %
v_p_: Blood plasma volume per unit volume of tissue, vp = CBV(1—Haematocrit), where CBV is cerebral blood volume, %
VIF_ICA_: Vascular input function (plasma concentration time course) derived from internal carotid artery just distal to the carotid syphon, mM
VIF_MCA_: Vascular input function (plasma concentration time course) derived from horizontal segment of the middle cerebral artery, mM
VOF_SSS_: Vascular output function (plasma concentration time course) derived from vertical/posterior third of the superior sagittal sinus, mM
VFA: Variable flip angle
VS: Vestibular schwannoma
